# Measurement invariance of the Functional Assessment of Cancer Therapy—Colorectal quality-of-life instrument among modes of administration

**DOI:** 10.1007/s11136-012-0272-x

**Published:** 2012-09-28

**Authors:** Carlos K. H. Wong, Cindy L. K. Lam, Brendan Mulhern, Wai-Lun Law, Jensen T. C. Poon, Dora L. W. Kwong, Janice Tsang

**Affiliations:** 1 Department of Family Medicine and Primary Care, The University of Hong Kong, 3/F, Ap Lei Chau Clinic, 161 Ap Lei Chau Main Street, Ap Lei Chau, Hong Kong Island, Hong Kong; 2 Health Economics and Decision Science, ScHARR, The University of Sheffield, Sheffield, UK; 3 Department of Surgery, The University of Hong Kong, Hong Kong, China; 4 Department of Clinical Oncology, The University of Hong Kong, Hong Kong, China

**Keywords:** Quality of life, FACT-C, Measurement invariance, Confirmatory factor analysis, Colorectal cancer, Mode of administration

## Abstract

**Objectives:**

To test for the measurement invariance of the Functional Assessment of Cancer Therapy—Colorectal (FACT-C) in patients with colorectal neoplasms between two modes of administration (self- and interviewer administrations). It is important to establish the measurement invariance of the FACT-C across different modes of administration to ascertain whether it is valid to pool FACT-C data collected by different modes or to assess each group separately.

**Methods:**

A cross-sectional sample of 391 Chinese patients with colorectal neoplasms was recruited from specialist outpatient clinics between September 2009 and July 2010. Confirmatory factor analysis (CFA) was used to test the original five-factor model of the FACT-C on data collected by self- and interviewer administrations in single-group analysis. Multiple-group CFA was then used to compare the factor structure between the two modes of administration using chi-square tests and other goodness-of-fit statistics.

**Results:**

The hypothesized five-factor model of FACT-C demonstrated good fit in each group. Configural invariance and metric invariance were fully supported in multiple-group CFA. Some item intercepts and their corresponding error variances were not identical between administration groups, suggesting evidence of partial strict factorial invariance.

**Conclusions:**

Our results confirmed that the five-factor structure of FACT-C was invariant in Chinese patients using both self- and interviewer administrations. It is appropriate to pool or compare data in the emotional well-being and colorectal cancer subscale scores collected by both administrations. Measurement invariance in three items, one from each of the other subscales, may be contaminated by response bias between modes of administration.

## Introduction

Health-related quality of life (HRQOL) is commonly used in clinical trials as an outcome measure in the evaluation of medical interventions. Most HRQOL measures are completed by self-administration using pencil and paper or interviewer administration using face-to-face or telephone-based approaches. Theoretically, interviewer administration has the advantage of feasibility (e.g., higher response rate), especially among subjects who have low literacy, manual dexterity problems or visual impairments. Trained interviewers can help patients in understanding ambiguous and complex item concepts with the support of standardized clarifications and explanations. However, completing HRQOL measures by self-administration eliminates interviewer bias and may save considerable time and costs [1].

Measurement invariance across groups can be assumed when the relationships between observed variables and latent variables are the same across samples. However, unless measurement invariance across subgroups has been demonstrated for an instrument, group comparisons of HRQOL scores collected by different modes of administration may be problematic [2–4]. Differences in the observed HRQOL scores may not truly reflect the real differences in the latent variables, but confounders and their interpretations may be biased, flawed or misleading. Therefore, it is important to establish evidence for invariance of measurement scores across groups to support fair and meaningful group comparisons of HRQOL outcome data [2]. There has been much research raising concerns about measurement invariance of HRQOL instruments across socio-demographic subgroups, such as gender [5–7], age [7–10] and ethnicity [11–16]. The effect of administration mode on HRQOL score has also been found in a number of cancer clinical trial studies using generic [17] or cancer-specific instruments [1, 11, 18, 19]. When differences in HRQOL scores are detected, the potential violation of measurement invariance across modes of administration needs to be considered. For example, a patient is hypothesized to express the same underlying level of HRQOL score through self- and interviewer administrations. Although both administration modes present the exact item and response wording, self-administration allows patients to reconsider and change their answers upon completion of the instrument. Interviewer administration may introduce barriers for patients to alter the answers of previous items. In addition, patients may tend to give more socially desirable responses in interviewer administration than in self-administration [20], responding in a socially favorable way of response. Therefore, not only are the observed variables given by the item responses related to latent variables, but they are also inferred by modes of administration.

The Functional Assessment of Cancer Therapy—General (FACT-G) is a commonly used HRQOL instrument in oncology. It was demonstrated to have a four-factor solution, corresponding to the original subscales, in the US population [1, 21], and later among Latin-American (e.g., Uruguay [22] and Colombia [23]) and UK [24] patients. The replication of the findings across countries provides strong evidence regarding the dimensionality of the instrument. Some of these studies were conducted using exploratory factor analysis, and the data-driven factor structure was found to differ from the hypothesized factor structure [23, 24]. Given the discrepancies between the data-driven and hypothesized factor structures, confirmatory factor analysis (CFA) was used to examine whether the data were the best fit with the hypothesized relationships of the instrument [1, 23]. The FACT—Colorectal (FACT-C), a colorectal cancer-specific HRQOL measure, is an extended version of the FACT-G. It comprises five subscales (the four FACT-G subscales and an additional concerns of “Colorectal Cancer Subscale”) [25]. The FACT instruments were principally designed for self-administration, but it can also be administered using interviews [26]. Measurement invariance of FACT-G between self- and interviewer administrations in patients with high literacy level has been illustrated [1]. However, the measurement invariance of the FACT has not been demonstrated between modes of administration in Chinese patients with a wider range of literacy competence.

The aims of the study were twofold. Firstly, we aimed to validate the conceptual measurement model of the FACT-C among Chinese patients with colorectal neoplasms. Secondly, we aimed to examine the measurement invariance of FACT-C scores collected using self- and interviewer administrations. Measurement invariance was assessed using sequential multiple-group CFA, a subcategory of modern quantitative approach [27]. The level of invariance of the FACT-C factor structure and factor loadings, and the equality of the item-level statistics across the two modes of administration groups were assessed. There are little data testing the factor structure and measurement model of the five-factor solution for the FACT-C using CFA. Furthermore, no studies have examined the factor structure of the FACT instruments in the Chinese population. To enable the combined analysis of FACT-C data collected by two different modes, measurement invariance should be established between them.

## Method

### Participants and data collection

A total of 647 patients were recruited from outpatient specialist colorectal clinics of a regional hospital in Hong Kong, China, between September 2009 and July 2010. All adults had a known diagnosis of colorectal neoplasms (colorectal polyp/cancer) for at least 6 months. In this study, colorectal polyp and colorectal cancer were defined as non-malignant and malignant tumors of the colon or the rectum or both, respectively. Patients completed the traditional Chinese version of the FACT-C and reported demographic variables such as age, education level, marital status, working status, smoking and drinking status, income and disease status. Of relevance to the current study, clinical variables and other HRQOL measures were also collected but were reported in previous papers [28–31]. In total, 57 subjects refused to participate in this study, and 41 subjects were excluded because they had a life expectancy of less than 6 months, were unable to understand and communicate in Chinese/Cantonese, displayed evidence of cognitive impairment or were too ill to participate in an interview. Subjects were allowed to choose their preferred instrument administration mode unless they could not complete the questionnaire by themselves. According to the FACT administration guideline [26], the face-to-face and telephone administrations were concurrently supported if adequate training was provided to each interviewer. At least two training sessions were given to each interviewer who was then instructed to go through each item of the questionnaire starting from the beginning to the end and to standardize how each item and its response options were read out during the interviews. Previous studies provided support for the use of FACT instruments by interviewer administration which was not partitioned into face-to-face and telephone interviews [1, 18]. Since then, 48 subjects by face-to-face and 108 subjects by telephone interviews were collapsed into one interviewer administration group in the current study to obtain a sufficient sample size. The aim of the study was explained to 549 eligible subjects (self-administration: 340; interviewer administration: 209), and written consent was obtained. Thirteen subjects withdrew from the study in the early part of survey, and 145 subjects did not complete the FACT-C component of survey. The remaining 391 subjects (self-administration: 235; interviewer administration: 156) with complete FACT-C data were included in the data analyses. Ethics approval was obtained from the Institutional Review Board of the University of Hong Kong and Hospital Authority, and the trial was registered with the HK Clinical Trial Register.

### The FACT-C

The FACT-C, the colorectal-specific module of the FACT measurement system [26], measures self-reported HRQOL during the past 7 days and has been extensively validated in English- [25], Spanish- [25], Korean- [32], French- [33] and Cantonese-speaking Chinese patients [28]. FACT-C aggregates 36 items into five dimensions: Physical Well-Being (PWB, 7 items), Functional Well-Being (FWB, 7 items), Social/Family Well-Being (SWB, 7 items), Emotional Well-Being (EWB, 6 items) and additional concerns of Colorectal Cancer Subscale (CCS, 9 items). Each item is rated by a five-point Likert scale (not at all, a little bit, somewhat, quite a bit, very much). FACT-C has been shown to have an acceptable degree of validity and reliability across a number of populations [25, 28, 32, 33] using classical quantitative approaches.

The FACT-C item related to sexual satisfaction (GS7, “I am satisfied with my sex life”) was omitted from analysis because of the conservative sexual attitude among Chinese society which resulted in a low overall response rate (38.8 %). Two items relating to ostomy appliances (C8, “I am embarrassed by my ostomy appliance”, and C9, “Caring for my ostomy appliance is difficult”) were not applicable to 251 colorectal cancer subjects without living with stoma, so they were omitted from the analyses.

### Data analysis

The patterns of socio-demographic and clinical characteristics of subjects with and without completion of FACT-C instrument were described. Independent *t* test and chi-square test were conducted to assess the differences between self-administered and interviewer-administered subjects. Descriptive analyses were carried out using SPSS 18.0 for Windows (SPSS Inc., Chicago, IL, USA).

#### Model estimation

Factor analyses were carried out using LISREL 8.80 program (Scientific Software International, Inc., Lincolnwood, IL, USA). The CFA models for ordinal data were performed using a polychoric correlation matrix to confirm the hypothesized factor structure for the FACT-C originally proposed by Ward et al. [25]. Diagonally weighted least-squares method, which is an estimator that can be used for ordinal data, was employed for parameter estimations. Missing data were excluded from mean comparisons and factor analysis.

#### Measurement invariance testing

Measurement invariance of the hypothesized five-factor model was tested individually using the self- and interviewer-administered data, and a combined model was also assessed. The importance of evaluating the factor structure of FACT-C in single-group analysis was to investigate whether the measurement model had five factors and whether the 33 items loaded on the same factor across each mode of administration. Multiple-group CFA was conducted to examine the extent of measurement invariance of the FACT-C factor structure across the mode of administration comparison groups, which were evaluated using four steps [2, 4, 8, 34, 35]. Firstly, configural invariance (which tests the equality of factor structures and model specification across groups) was used to assess whether the hypothesized five-factor model is the same across groups. If there is configural invariance between the models, it is unnecessary to perform subsequent analyses of measurement invariance. Secondly, metric invariance (which tests equality of factor loadings across groups) was examined by constraining the factor loadings to be equal across groups. Thirdly, scalar invariance (which tests equality of item intercepts across groups) was examined. Scalar invariance is satisfied if the item intercepts and factor loadings are constrained to be identical across groups. Finally, strict factorial invariance (which tests the equality of item residuals across groups) was examined. Strict factorial invariance is achieved only if configural invariance, metric invariance, scalar invariance and item residuals are constrained to be equal across groups simultaneously [2]. The analytic procedures applied a “step-up” strategy that began with unconstrained model and consecutively restricted constrained models [35]. In each level of invariance testing, partial measurement invariance was assessed using the conventional Cheung and Rensvold [36] approach to determine whether the removal of cross-group constraints would improve the model fit substantially after re-specification of models. This approach was initially developed for testing partial metric invariance (partial equality of factor loadings across groups), but it can be applied to assess partial scalar invariance (partial equality of item intercepts across groups) and strict factorial invariance (partial equality item residuals across groups) [4].

#### Goodness-of-fit statistics

The model goodness-of-fit statistics were primarily assessed using root mean square error of approximation (RMSEA) [37], comparative fit index (CFI) [38] and Tucker–Lewis index (TLI). Besides these absolute and incremental fit measures, the Satorra–Bentler scaled chi-square statistic (SB χ^2^) [39] was estimated using the diagonally weighted least-squares method with degrees of freedom (*df*) reported to reflect the model fit. CFA Models were considered to have acceptable model fit if RMSEA values and their 90 % confidence intervals were close to 0.08 or below and CFI and TLI values were close to 0.95 or greater [40]. For multiple-group comparisons, the Satorra–Bentler scaled chi-square difference (∆SB χ^2^) test and the change in CFI (∆CFI) [41] were used to compare the model fit of the more constrained model with that of the less constrained model. *P* value of <0.05 was considered statistically significant for ∆SB χ^2^ test, implying the null hypothesis of invariance (or constrained model) should not be rejected. The ∆CFI of >−0.01 was recommended to indicate invariance [41].

## Results

### Sample characteristics

Table 1 shows the socio-demographic and clinical characteristics of patients in overall, by administration modes and by completion of FACT-C instrument. The mean age of interviewer-administered patients was significantly higher than that of the self-administered patients (66.0 ± 12.0 vs. 61.2 ± 10.8, *P* < 0.001). Self-administered patients were more likely to have younger age, received at least primary school education (*P* < 0.001), work (*P* = 0.001) or more income (*P* < 0.001) than interviewer-administered patients. CRC patients were more likely to be interviewer-administered than to be self-administered (*P* = 0.041). Among CRC patients, those who were on palliative treatment (*P* = 0.007) or had stoma (*P* = 0.045) were associated with interviewer administration.

**Table 1:** Socio-demographic characteristics of study subjects by mode of administration and by completion of FACT-C instrument

	Self (*n* = 235)	Interview (*n* = 156)	Complete		Incomplete	
			Total (*n* = 391)	*P* value^†^	Total (*n* = 145)	*P* value^‡^
Age (year, mean ± SD)^†‡^	61.2 ± 10.8	66.0 ± 12.0	63.1 ± 11.5	<0.001	65.9 ± 9.8	0.009
Sex (%)				0.697		0.098
Male	59.6	61.5	60.4		52.4	
Female	40.4	38.5	39.6		47.6	
Education level (%)^†^				<0.001		0.102
No formal school	7.2	17.3	11.3		10.4	
Primary	28.9	37.8	32.5		42.4	
Secondary	45.5	36.5	41.9		38.9	
Tertiary	18.3	8.3	14.3		8.3	
Marital status (%)^‡^				0.431		<0.001
Married	82.1	78.8	80.8		60.0	
Not married	17.9	21.2	19.2		40.0	
Currently working (%)^†^				0.001		0.183
Yes	28.9	14.7	23.3		17.9	
No	71.1	85.3	76.7		82.1	
Income (HKD, %)^†‡^				<0.001		<0.001
≤$20,000	72.6	92.9	80.7		94.3	
>$20,000	27.4	7.1	19.3		5.7	
Smoking (%)				0.366		0.194
Ever had	26.0	30.1	27.6		22.1	
Never had	74.0	69.9	72.4		77.9	
Drinking (%)				0.326		0.436
Ever had	30.2	25.6	28.4		25.0	
Never had	69.8	74.4	71.6		75.0	
Colorectal neoplasm (%)^†‡^				0.041		0.049
CRC	70.2	79.5	73.9		82.1	
Polyps	29.8	20.5	26.1		17.9	
Active CRC treatment (%)*^†^	*n* = 165	*n* = 124	*n* = 289	0.007	*n* = 119	0.403
No	77.6	71.8	75.1		79.8	
Adjuvant	10.3	4.0	7.6		4.2	
Palliative	12.1	24.2	17.3		16.0	
Stoma (%)*^†^	*n* = 165	*n* = 124	*n* = 289	0.045	*n* = 119	0.456
Present	9.7	17.7	13.1		16.0	
Absent	90.3	82.3	86.9		84.0	

*CRC* colorectal cancer

* Colorectal cancer (CRC) group only

^†^Significant difference between modes of administration by chi-square or independent *t* test

^‡^Significant difference between subjects completing and not completing FACT-C by chi-square or independent *t* test

### FACT-C descriptive statistics

Table 2 shows the mean, standard deviation, and floor and ceiling effects of items among all patients, and separately for the self-administration and interviewer administration groups. Overall, three out of seven CCS items, all PWB items and five out of six EWB items had floor effect, defined as floor percentage >30 %. Ceiling effects (>30 %) were observed in four out of six SWB items. Table 3 shows the mean differences in FACT-C subscale scores with and without adjustments for socio-demographic and clinical characteristics. Statistical differences between self- and interviewer administrations were found in PWB, EWB and CCS subscale scores (*P* < 0.001, *P* < 0.001, *P* < 0.001). Adjusted results of those subscale scores were also significantly different between modes of administration (PWB, *P* < 0.001, EWB, *P* < 0.001, CCS, *P* = 0.002). Both the unadjusted and adjusted differences in those subscale scores were negative, indicating that interviewer administration had higher estimated HRQOL than self-administration.

**Table 2:** Descriptive statistics of 33 items of the FACT-C among two modes of administration

Item	Interview (*n* = 156)				Self (*n* = 235)				Total (*n* = 391)				*P* value^†^
	Mean	SD	Floor %	Ceiling %	Mean	SD	Floor %	Ceiling %	Mean	SD	Floor %	Ceiling %	
PWB^†^	25.36	3.31			23.06	4.95			23.97	4.51			<0.001
GP1^†^	0.59	0.91	63	1	1.00	1.11	43	3	0.84	1.05	51	2	<0.001
GP2^†^	0.12	0.39	90	0	0.42	0.80	74	0	0.30	0.68	81	0	<0.001
GP3^†^	0.12	0.45	92	0	0.73	1.00	55	3	0.48	0.88	70	2	<0.001
GP4^†^	0.51	0.85	69	0	0.72	0.89	51	1	0.64	0.88	58	1	0.020
GP5^†^	0.33	0.81	82	1	0.83	1.09	55	3	0.63	1.02	66	2	<0.001
GP6^†^	0.64	0.92	60	1	0.84	0.98	48	1	0.76	0.96	52	1	0.042
GP7	0.33	0.76	79	0	0.41	0.84	76	0	0.38	0.81	77	0	0.368
SWB	19.89	4.72			20.05	6.14			19.99	5.61			0.776
GS1*	2.46	1.04	8	12	2.61	1.13	6	23	2.55	1.09	7	18	0.194
GS2*	3.03	0.84	3	26	3.09	0.99	2	40	3.06	0.93	2	35	0.582
GS3*	2.60	1.06	8	15	2.72	1.13	4	27	2.67	1.10	6	22	0.263
GS4*	3.13	0.75	2	28	3.23	0.99	3	50	3.19	0.90	2	41	0.330
GS5*	3.06	0.87	3	28	3.13	0.97	3	41	3.10	0.93	3	36	0.466
GS6*	2.97	0.96	5	28	3.05	1.10	5	42	3.02	1.05	5	36	0.479
EWB^†^	21.12	3.15			18.73	4.66			19.68	4.29			<0.001
GE1^†^	0.38	0.78	76	0	0.75	0.99	54	2	0.61	0.93	63	1	<0.001
GE2*^†^	3.05	0.84	3	28	2.74	1.13	6	28	2.87	1.03	5	28	0.004
GE3^†^	0.26	0.74	86	1	0.43	0.79	71	1	0.37	0.78	77	1	0.032
GE4^†^	0.32	0.68	78	0	0.89	1.05	44	4	0.66	0.96	58	2	<0.001
GE5^†^	0.27	0.70	84	1	0.74	1.01	57	3	0.55	0.93	68	2	<0.001
GE6^†^	0.69	0.99	61	1	1.20	1.18	35	6	0.99	1.13	45	4	<0.001
FWB	18.87	4.73			18.54	6.47			18.67	5.83			0.583
GF1*^†^	2.27	1.27	13	17	2.63	1.17	8	26	2.49	1.22	10	23	0.004
GF2*	2.81	0.96	4	21	2.63	1.16	6	25	2.71	1.09	6	23	0.109
GF3*	2.76	0.81	3	12	2.73	1.10	4	27	2.74	1.00	3	21	0.812
GF4*^†^	3.16	0.70	2	28	2.80	1.03	4	26	2.94	0.93	3	27	<0.001
GF5*	2.45	1.04	6	13	2.41	1.15	8	18	2.43	1.11	7	16	0.754
GF6*	2.57	1.05	7	15	2.65	1.11	6	24	2.62	1.08	6	20	0.496
GF7*	2.85	0.82	3	17	2.69	1.09	4	25	2.75	0.99	4	21	0.112
CCS^†^	21.78	3.61			20.10	5.05			20.77	4.60			<0.001
C1^†^	0.36	0.73	76	0	0.76	0.93	51	1	0.60	0.88	61	1	<0.001
C2	0.32	0.70	78	1	0.44	0.81	71	1	0.39	0.77	74	1	0.139
C3*	2.51	1.17	11	17	2.28	1.35	16	20	2.37	1.28	14	19	0.075
C4*	2.77	0.88	3	15	2.58	1.10	6	20	2.65	1.02	5	18	0.071
C5^†^	0.36	0.67	74	0	0.71	1.00	57	2	0.57	0.90	64	1	<0.001
C6*	2.80	0.84	2	16	2.71	1.18	6	29	2.75	1.06	5	24	0.408
C7*^†^	2.73	0.95	4	19	2.44	1.18	9	20	2.56	1.11	7	20	0.011

* Higher item scores indicate better HRQOL; for other items, higher scores indicate worse HRQOL

^†^Significant difference between interviewer and self-administrations by independent *t* test

**Table 3:** Unadjusted and adjusted mean differences in FACT-C subscales between modes of administration

	Mean difference (Self- interview)					
	Unadjusted			Adjusted^‡^		
	Mean	95 % CI	*P* value	Mean	95 % CI	*P* value
PWB*^†^	−2.30	(−3.19, −1.42)	<0.001	−2.26	(−3.27, −1.26)	<0.001
SWB	0.17	(−0.98, 1.31)	0.776	0.49	(−0.73, 1.71)	0.432
EWB*^†^	−2.39	(−3.23, −1.56)	<0.001	−2.20	(−3.14, −1.25)	<0.001
FWB	−0.33	(−1.52, 0.85)	0.583	−0.25	(−1.57, 1.07)	0.707
CCS*^†^	−1.67	(−2.59, −0.75)	<0.001	−1.72	(−2.78, −0.65)	0.002

*Significant difference between modes of administration by independent *t* test

^†^Significant difference between modes of administration by regression

^‡^Mean differences were adjusted by socio-demographic and clinical characteristics by regression

### Factor structure

Table 4 demonstrates the goodness-of-fit indices of two CFA models in single-group analysis, overall and separately on data collected by two modes of administration. For those items with negative response choices, the responses were reversed to achieve consistency in positive factor loadings. In single-group analyses of self- and interviewer-administered data, factor loadings of all items except C5 (“I have diarrhea (diarrhoea)”) exceeded 0.4, achieving substantial interpretability of underlying factor structure. Based on the conventional guidelines [40], the original five-factor CFA model met all of the fit criteria for data by self-administration (RMSEA = 0.072, 90 % CI = 0.066–0.077, CFI = 0.977, TLI = 0.975) and passed the criteria for data by interviewer administration (RMSEA = 0.065, 90 % CI = 0.057–0.073, CFI = 0.963, TLI = 0.960). The CFI and TLI were significantly better in self-administered data than in interviewer-administered data.

**Table 4:** Factor loadings among two modes of administration in single-group analyses

Subscale/item	Interview (*n* = 156)*		Self (*n* = 235)^†^		Total (*n* = 391)^‡^	
	Factor loading	*R* ^2^	Factor loading	*R* ^2^	Factor loading	*R* ^2^
PWB						
GP1	0.654	0.427	0.716	0.512	0.708	0.501
GP2	0.653	0.426	0.700	0.491	0.719	0.516
GP3	0.793	0.628	0.749	0.562	0.769	0.592
GP4	0.520	0.270	0.723	0.523	0.657	0.431
GP5	0.646	0.418	0.770	0.592	0.765	0.585
GP6	0.701	0.491	0.902	0.814	0.831	0.690
GP7	0.870	0.756	0.881	0.776	0.853	0.727
SWB						
GS1	0.792	0.627	0.866	0.751	0.836	0.699
GS2	0.864	0.746	0.915	0.837	0.896	0.804
GS3	0.840	0.705	0.859	0.739	0.838	0.703
GS4	0.845	0.714	0.875	0.766	0.848	0.720
GS5	0.831	0.691	0.891	0.794	0.881	0.777
GS6	0.724	0.524	0.827	0.684	0.801	0.642
EWB						
GE1	0.876	0.767	0.812	0.659	0.836	0.698
GE2	0.678	0.460	0.733	0.537	0.708	0.502
GE3	0.842	0.709	0.904	0.816	0.891	0.793
GE4	0.615	0.379	0.807	0.652	0.781	0.609
GE5	0.764	0.584	0.743	0.553	0.780	0.608
GE6	0.632	0.400	0.837	0.700	0.803	0.644
FWB						
GF1	0.602	0.363	0.837	0.701	0.706	0.498
GF2	0.735	0.541	0.917	0.842	0.859	0.737
GF3	0.843	0.711	0.902	0.814	0.883	0.780
GF4	0.792	0.627	0.785	0.616	0.791	0.626
GF5	0.659	0.434	0.755	0.569	0.716	0.513
GF6	0.672	0.451	0.894	0.799	0.814	0.662
GF7	0.802	0.644	0.869	0.755	0.848	0.719
CCS						
C1	0.715	0.511	0.651	0.424	0.689	0.474
C2	0.627	0.393	0.544	0.296	0.572	0.328
C3	0.467	0.218	0.690	0.476	0.623	0.388
C4	0.608	0.370	0.730	0.532	0.690	0.477
C5	0.303	0.092	0.398	0.159	0.412	0.170
C6	0.524	0.275	0.791	0.625	0.705	0.496
C7	0.758	0.575	0.818	0.668	0.787	0.620

*Goodness-of-fit of CFA model by self-administration: RMSEA = 0.072, 90 % CI = 0.066–0.077, CFI = 0.977, TLI = 0.975

^†^Goodness-of-fit of CFA model by interviewer administration: RMSEA = 0.065, 90 % CI = 0.057–0.073, CFI = 0.963, TLI = 0.960

^‡^Goodness-of-fit of CFA model: RMSEA = 0.068, 90 % CI = 0.063–0.072, CFI = 0.975, TLI = 0.973

### Multiple-group CFA

Table 5 shows the results of the single-group and multiple-group CFA for testing invariance between modes of administration. Multiple-group CFA initially started with the assessment of configural invariance with five-factor model. Model 3 precluded the equality constraints on parameter estimates across modes of administration, and the model fitted well (RMSEA = 0.0665, CFI = 0.962, TLI = 0.958) in two groups, supporting evidence of full configural invariance (i.e., the hypothesized factor model was equivalent across groups).

**Table 5:** Model fit indices summary for single-group and multiple-group comparisons

Model type	Constraints	SB χ2	*df*	RMSEA	RMSEA (90 % CI)	CFI	TLI	Model comparison	∆SB χ2	∆*df*	*P* value	∆CFI
*Factor structure in single group*												
1. Interviewer administration	None	799.64	485	0.0647	(0.0566, 0.0726)	0.963	0.960	NA	NA	NA	NA	NA
2. Self-administration	None	1066.81	485	0.0716	(0.0658, 0.0774)	0.977	0.975	NA	NA	NA	NA	NA
*Multiple-group comparisons*												
3. Full configural invariance	Factor structure	1804.21	970	0.0665	(0.0617, 0.0712)	0.962	0.958	NA	NA	NA	NA	NA
4. Full metric invariance	Factor structure, factor loadings	1837.51	998	0.0658	(0.0610, 0.0705)	0.962	0.959	3 vs 4	33.29	28	0.225	0.000
5. Full scalar invariance	Factor structure, factor loadings, intercepts	1964.54	1026	0.0686	(0.0640, 0.0731)	0.957	0.956	3 vs 5	127.04	28	0.000	-0.005
6. Partial scalar invariance	Factor structure, factor loadings, intercepts (GF1, GP3, GS3 freely estimated)	1862.34	1023	0.0649	(0.0603, 0.0696)	0.962	0.960	4 vs 6	24.83	25	0.472	0.000
7. Partial strict factorial invariance	Factor structure, factor loadings, intercepts (GF1, GP3, GS3 freely estimated), error variance (GF1, GP3, GS3 freely estimated)	1839.99	1053	0.0620	(0.0573, 0.0667)	0.964	0.964	6 vs 7	22.35	30	0.841	0.002

*SB* Satorra–Bentler, *df* degree of freedom, *RMSEA* root mean square error of approximation, *CFI* comparative fit index, *TLI* Tucker–Lewis index

The following model (Model 4) tested metric invariance by imposing additional equality constraint on the factor loadings. An acceptable fit of Model 3 to the data was significantly better than (∆SB χ^2^ = 33.29, ∆*df* = 28, *P* value = 0.225, ∆CFI = 0.000) that for Model 4, which further supported the evidence of full metric invariance. In other words, the factor loadings were numerically identical in both administration groups.

Model 5 tested full scalar invariance by imposing the equality constraint on the intercepts. Full scalar invariance across groups was not fully supported as indicated by a statistically significant misfit (∆SB χ^2^ = 127.04, ∆*df* = 28, *P* value < 0.001, ∆CFI = −0.005). Upon rejection of full scalar invariance, separate CFA models were tested to allow the intercept of specific items to be freely estimated in order to test for partial scalar invariance. Model 6 presented the improvement in model fit when allowing intercepts of item GP3 (“Because of my physical condition, I have trouble meeting the needs of my family”), GS3 (“I get support from my friends”) and GF1 (“I am able to work (include work at home)”) to vary. The change in goodness-of-fit was good for Model 6 (RMSEA = 0.0649, CFI = 0.962), as compared to the full scalar invariance model (Model 5). Furthermore, compared with full metric invariance model (Model 4), Model 6 reported a better model fit (∆SB χ^2^ = 24.83, ∆*df* = 25, *P* value = 0.472, ∆CFI = 0.000), suggesting partial scalar invariance (i.e., intercepts of all items except GP3, GS3 and GF1 were equivalent across groups) between self-administration and interviewer administration groups.

The final step in our invariance analyses tested partial strict factorial invariance by additionally restricting the error variances specific to item GP3, GS3 and GF1 to be equal among groups. Our results indicated better model fit in Model 7 (∆SB χ^2^ = 22.35, ∆*df* = 30, *P* value = 0.841, ∆CFI = 0.002) when compared with the former partial scalar invariance model (Model 6). As a whole, our results indicated retention of partial strict factorial invariance between administration groups.

## Discussion

This study examined the factor structures and measurement invariance of FACT-C between two administration groups. Our results showed significant mean differences in the physical and emotional well-being and colorectal-specific scores of the FACT-C between administration modes, while the differences were previously driven by lower estimated scores among self-administration rather than among interviewer administration on physical and emotional subscale scores of the FACT-G [1]. No difference was found in the social and functional subscale scores between administration modes. Differentials in administration modes were not significantly reduced by adjustment for socio-demographic and clinical factors. Unlike previous studies [1, 11, 19] that examined the relationship between factors and FACT-G subscale scores, mode of administration was an insignificant determinant of physical and emotional subscale scores in this study. Interviewer-administered patients did not report significantly lower HRQOL in social and functional subscale scores when compared with self-administered patients, which is in contrast to findings of previous studies [1, 11, 19].

Our CFA results provided empirical evidence to support the hypothesized five-factor structure and conceptual measurement model of the FACT-C [25]. It showed a satisfactory model fit without the need of any modification in the hypothesized factor structure in our Chinese colorectal neoplasms patients, irrespective of whether FACT-C was self-administered or interviewer-administered. In a previous study that examined the FACT-G in a Latin-American sample [23], modification of the original four-factor structure was suggested by factor analysis, with minor modifications to the emotional subscale. Factor analysis conducted in a UK sample [24] found that some items in the social and functional subscales did not load satisfactorily (*r* < 0.5) on any existing factors, and this indicated potential ambiguity in the measurement of the underlying constructs. In contrast, we found that the factor loadings of all items in the physical, social, emotional and functional subscales were satisfactorily (*r* ≥ 0.5) correlated with their hypothesized factors within the colorectal-specific FACT-C measure. In the last seven items concerning colorectal cancer, five loaded on the colorectal specific factor.

Our multiple-group CFA supported full metric invariance (Model 5) which implied that the factor structure and corresponding factor loadings were equal between the two modes of administration. However, three violations of scalar invariance were identified in three different items in regard to mode of administration. The hypothesis of full scalar invariance was not supported because the FACT-C instrument was not invariant in intercepts in GP3 (“Because of my physical condition, I have trouble meeting the needs of my family”), GS3 (“I get support from my friends”) and GF1 (“I am able to work (include work at home)”) of the physical, social and functional subscales, respectively, between administration groups. Partial scalar invariance implied that group differences observed in the physical, social and functional subscales were not interpreted to reflect the real differences in the particular constructs. In other words, physical, social and functional subscales were measured similarly across administration groups when employing the hypothesized five-factor model of the FACT-C measure. Current findings established that the mean comparisons of those subscale scores differ between groups that used different modes of administration, particularly conveying a message that it is appropriate to pool or compare the data in emotional well-being and colorectal cancer subscale scores collected by two administration groups. In general, one possible explanation for the variance is the response bias introduced by the differential effects of administration mode [4]. Interviewer administration has a tendency to lead to responses in line with acceptable social norms, the so-called social desirability bias [20]. Cancer patients were found to inflate their HRQOL and report more socially favorable and desirable responses when the instrument was administered by an interviewer in a randomized study of administration modes [17]. Concerning the measurement non-invariance in three individual items (GP3, GS3 and GF1), patients may interpret diverse understanding from those items across administration modes or mixed up with other items that carry similar concept at the interviews. For example, most patients (76.3 %) held the same answer for item GS3 (“I get support from my friends”) and GS1 (“I feel close to my friends”) in interviewer administration, but it was less likely to occur (69.4 %) in self-administration. Evidence of the socio-demographics and diseases measurement invariance of the FACT-C across groups should be provided in further studies. It is certainly worthwhile to consider the measurement invariance of the FACT-C in relation to other patient characteristics because differentials in latent HRQOL may exist in those characteristics.

Findings of measurement non-invariance in factor structure, loading or intercepts call for caution in interpreting group differences between self-administered and interviewer-administered FACT instruments Removing misfit items is recommended to improve the measurement properties of FACT instruments [1, 24]. For instance, Hahn et al. [1] did not identify misfit items in the PWB and SWB subscales of the FACT-G English version and PWB and FWB subscales of the Spanish version. Mean comparisons of the misfit items and corresponding subscales between groups might be out of the scope of this study. Further studies should apply contemporary psychometric methods such as item-response theory and Rasch analysis to investigate the factor structure and psychometric performance of the FACT-C scales.

### Limitations

Firstly, our study results were based on a convenience sample of Chinese patients with colorectal neoplasms, which did not necessitate the generalizability to non-Chinese or other Chinese populations. Investigations into measurement invariance in other cultural or ethnic groups of patients with colorectal neoplasms are needed. Secondly, the removal of the sensitive item related to sex and the items related to stoma that had low response rates limits the applicability of the results to these items. Further empirical evaluations of data from patients who are more likely to answer the sexual activity item may be useful to overcome this shortcoming. Finally, there were significant differences in socio-demographic characteristics between the self-administration and interviewer administration groups, possibly due to own preference for administration modes in the outpatient setting, which could have introduced measurement variance in addition to the mode of administration. Given the patient preferences in decision making for the administration modes in the current study, younger or lower-disease-severity patients preferred self-administration to interviewer administration except those patients who had literacy dexterity or visual difficulty, and only interviewer administration was feasible. To eliminate the bias, patients’ characteristics should be well balanced across the two administration groups when randomization is conducted in a further study.

## Conclusion

Our results revealed that the five-factor structure of FACT-C provided excellent fit when the HRQOL instrument was either self-administered or interviewer-administered, separately or simultaneously. The construct validity of FACT-C was supported in Chinese patients with colorectal neoplasms. Given the acceptable degree of cross-group measurement invariance, it is valid to compare EWB and CCS scores collected by these two modes of administration. Complete measurement invariance could be contaminated by response bias by administration modes in other subscales. Pooling or direct comparison of FACT-C data collected by a mixture of self- and interviewer administrations should be done with caution.

## Abbreviations

HRQOL: Health-related quality of life
FACT-G: Functional Assessment of Cancer Therapy—General
FACT-C: Functional Assessment of Cancer Therapy—Colorectal
CFA: Confirmatory factor analysis
PWB: Physical Well-Being
FWB: Functional Well-Being
SWB: Social/Family Well-Being
EWB: Emotional Well-Being
CCS: Colorectal Cancer Subscale
DWLS: Diagonally weighted least square
RMSEA: Root mean square error of approximation
CFI: Comparative fit index
TLI: Tucker–Lewis index
SB χ^2^: Satorra–Bentler scaled chi-square statistic
*df*: Degrees of freedom
